# Pollinator species richness and abundance across diverse habitat-types on Terceira Island (Azores, Portugal)

**DOI:** 10.3897/BDJ.13.e142482

**Published:** 2025-01-22

**Authors:** Mário Boieiro, Raúl Oliveira, Ricardo Costa, Paulo A. V. Borges

**Affiliations:** 1 LIBRe – Laboratory for Integrative Biodiversity Research, Finnish Museum of Natural History, University of Helsinki, Helsinki, Finland LIBRe – Laboratory for Integrative Biodiversity Research, Finnish Museum of Natural History, University of Helsinki Helsinki Finland; 2 University of Azores, CE3C—Centre for Ecology, Evolution and Environmental Changes, Azorean Biodiversity Group, CHANGE —Global Change and Sustainability Institute, School of Agricultural and Environmental Sciences, Rua Capitão João d’Ávila, Pico da Urze, 9700-042, Angra do Heroísmo, Azores, Portugal University of Azores, CE3C—Centre for Ecology, Evolution and Environmental Changes, Azorean Biodiversity Group, CHANGE —Global Change and Sustainability Institute, School of Agricultural and Environmental Sciences, Rua Capitão João d’Ávila, Pico da Urze, 9700-042 Angra do Heroísmo, Azores Portugal; 3 IUCN SSC Atlantic Islands Invertebrate Specialist Group, Angra do Heroísmo, Azores, Portugal IUCN SSC Atlantic Islands Invertebrate Specialist Group Angra do Heroísmo, Azores Portugal; 4 Mestrado em Gestão e Conservação da Natureza, University of the Azores, Angra do Heroísmo, Azores, Portugal Mestrado em Gestão e Conservação da Natureza, University of the Azores Angra do Heroísmo, Azores Portugal; 5 IUCN SSC Monitoring Specialist Group, Angra do Heroísmo, Azores, Portugal IUCN SSC Monitoring Specialist Group Angra do Heroísmo, Azores Portugal

**Keywords:** alien species, island endemics, generalist species, intensive pastures, pan traps, semi-natural pastures, transect sampling

## Abstract

**Background:**

Azorean biodiversity is relatively well-known following important scientific contributions during the last three decades. These have set a comprehensive species checklist for the Archipelago, improved significantly the knowledge on species abundance, ecology and distribution and have contributed to define priorities for conservation management and scientific research. Nevertheless, despite these efforts, a key functional group - the pollinators - remains poorly known in Azores, including their occurrence in different habitat-types and islands. Insect pollinators play a key ecological role and a valuable ecosystem service being crucial to having basic information on their abundance, distribution and ecology and a good knowledge on the status of their populations, if we aim to ensure the long-term sustainability of terrestrial ecosystems. Furthermore, island ecosystems are facing significant pressures from land-use and climatic changes and, from the increasing arrival of alien species to these remote areas, presenting a pressing need to assess the effects of these factors on island pollinators and pollination.

**New information:**

Here, we present an inventory of the pollinator species found in different habitat-types of Terceira along a gradient of disturbance and encompassing 30 sites distributed throughout the island. We identified 2547 pollinators from 40 taxa, mostly dipterans and hymenopterans and recorded novel information on species distribution and ecological associations. A high number of taxa are native species, including three Azorean endemics, but 14 species are alien to the Archipelago. The use of a combination of standardised sampling techniques allowed us to collect information on diverse pollinator groups, but, most importantly, the data collected will contribute to assess the impacts of human activities on pollinator abundance and richness and support decision-making on habitat management for pollinators in the Azores.

## Introduction

Pollinators play a fundamental role on the sustainability of most terrestrial ecosystems and provide a vital ecosystem service to human-kind through increased agricultural production and enhanced food security (Ollerton 2021). The large majority of flowering plant species depends, to some degree, on animal pollination services and over 75% of the major global-scale food crops rely on pollinators for yield and/or quality (Klein et al. 2007, Ollerton et al. 2011). On island ecosystems, pollinators are also known to play important roles on biodiversity conservation, agricultural production and human well-being. For instance, several studies have identified pollinator species/groups that are important flower visitors and may influence directly or indirectly the reproduction and survival of island endemic plants (Valido et al. 2002, Jaca et al. 2019, Esposito et al. 2021, Hazlehurst et al. 2023). Additionally, other studies have assessed the socioeconomic relevance of ecosytem services to island societies, highlighting the role of pollination in agricultural production and the local economic gains (Picanço et al. 2017a, Balzan et al. 2018a, Balzan et al. 2018b).

Island pollinators are threatened by the same factors that affect their continental counterparts, particularly land-use change leading to habitat loss and fragmentation, environmental pollution (including pesticide application), alien species and climate change (Potts et al. 2010, Vanbergen 2013, IPBES 2016, Brunet and Fragoso 2024 and references therein). However, the effects of these factors on island pollinator species are usually more severe due to their restricted distributions, smaller and fewer populations, but also because many species are more vulnerable to the effects of human disturbance and alien species as a result of their evolution in isolation (Whittaker and Fernández-Palacios 2007). A recent global analysis of the response of pollinator biodiversity to land-use type and management intensity showed reductions in species richness and abundance, but only at high land-use intensities (Millard et al. 2021). Nevertheless, significant changes in species composition in favour of more generalist species have often been reported as a consequence of human disturbance or transformation of natural habitats (e.g. Biella et al. (2022), Ren et al. (2022)). The invasion of island ecosystems by alien plants and pollinators is also a matter of great concern (Morales and Aizen 2002, Hansen et al. 2018). Alien plants and pollinators can drastically change natural plant-pollinator interactions, eventually leading to population reductions and local extinctions of native pollinators (Kaiser-Bunbury et al. 2011). In the Azores, there has been a continuous and alarming increase in the number of plant and animal species introductions, including some pollinators, but the effects of this issue on ecological processes and native biodiversity remain unknown (Borges et al. 2013, Borges et al. 2020, Borges et al. 2022b, Boieiro et al. 2023, Boieiro et al. 2024b). Previous and ongoing monitoring and conservation projects targeting the Azorean terrestrial invertebrates have been important in gathering baseline information on pollinators and have also indirectly benefitted pollinator populations through programmed native habitat restoration initiatives (e.g. Lhoumeau et al. (2024), Pozsgai et al. (2024)).

## General description

### Purpose

The goal of this study is to provide data on the abundance and diversity of pollinators from 30 sites distributed by three different habitat-types (intensive pastures, semi-natural pastures and naturalised vegetation) in Terceira Island (Azores, Portugal). We focused on the insect groups considered the most important pollinators (e.g. bees, beetles, butterflies, hoverflies) and we have used a combination of complementary sampling techniques (standardised observation transects, pan traps and vegetation sweeping).

## Project description

### Title

Pollinator abundance and diversity in different habitat-types of Terceira Island (Azores, Portugal).

### Personnel

Fieldwork (site selection and experimental setting): Mário Boieiro and Paulo A.V. Borges.

Fieldwork (authorisation): Azorean Regional Government, Science and Technology Directorate - Internationally Recognised Compliance Certificate - CCIR-RAA/2023/28.

Fieldwork (sample collection): Raúl Oliveira and Mário Boieiro.

Parataxonomist: Raúl Oliveira.

Taxonomists: Mário Boieiro, Ricardo Costa and Paulo A. V. Borges.

Voucher specimen management: Raúl Oliveira.

Database management: Raúl Oliveira, Mário Boieiro and Paulo A. V. Borges.

Darwin Core databases: Mário Boieiro and Paulo A. V. Borges.

### Funding

Main funding for research and fieldwork was obtained from FCT-UIDB/00329/2020-2024 DOI 10.54499/UIDB/00329/2020 (Thematic Line 1 – integrated ecological assessment of environmental change on biodiversity) and Azores DRCT Pluriannual Funding (M1.1.A/FUNC.UI&D/010/2021-2024). Data curation and open access of this manuscript were supported by the project: FCT-UIDB/00329/2020-2024 DOI: 10.54499/UIDB/00329/2020.

## Sampling methods

### Study extent

The study was carried out using a combination of standardised sampling techniques, namely observation transects, pan traps and vegetation sweeping (Fig. 1). These methods provide complementary information and their use in combination is often recommended (Roulston et al. 2007, Popic et al. 2013, O'Connor et al. 2019). The methodology we followed is similar to that adopted by the European project SPRING - Strengthening Pollinator Recovery through INdicators and monitoringinG (https://pollinator-monitoring.net), under the EU Pollinator Monitoring Scheme (EU PoMS), with the necessary adaptations to match our specific goals (see below).

### Sampling description

In this study, we applied three sampling techniques, namely direct observations of pollinators along 50-metre linear transects, pan trapping and vegetation sweeping. Sampling was carried out in the 30 study sites during sunny or partially cloudy days, without rain and with little or no wind, as these are the best conditions for observing pollinators (Pollard and Yates 1993, Herrera 1997). Transect sampling was carried out along 50-metre linear transects, 2 metres wide (100 m^2^/site) between 10.00 AM and 4.00 PM. The transects were walked at a regular pace and the pollinators found on flowers or flying were identified on the spot, whenever possible to species level or captured to confirm taxonomic identification in the laboratory. Pan trapping consisted of placing two sets of traps in each site, approximately 50 m apart, one at the beginning and the other at end of the transect. Pan-traps are colourful traps aimed to attract and capture pollinators and should be active during insect peak activity. Each set of traps combined three plastic containers (11 cm in diameter) of different colours (blue, white and yellow) that were used to optimise the capture of different groups of pollinators (Vrdoljak and Samways 2012). The traps were filled with water with a few drops of detergent and were fixed at a height corresponding to the average height of the flowers at each sampling point. The pan traps remained in place between 9.00 AM and 6:00 PM and the insects were subsequently collected and transported to the laboratory. The vegetation sweeping consisted in sampling the vegetation along 50-m transects using an entomological net (36 cm in diameter), allowing the collection of cryptic pollinator species. This technique was also carried out in all transect sampling sites. The collected insects were placed in vials with ethanol (96%) for later identification in the laboratory.

### Quality control

During transect sampling, many individuals (e.g. butterflies and hoverflies) were identified to species level on the spot due to their characteristic morphological features. However, most individuals were collected with the help of an entomological net, labelled and stored in vials with ethanol (96%). The samples with specimens collected in pan traps and captured by sweeping were labelled, stored in vials with ethanol and transported to the laboratory. In the laboratory, the specimens of the target groups were sorted and identified to species level with the help of a stereomicroscope (Leica S9i) and using specific literature (Rojo et al. 1997, Amiet et al. 2001, Gereys 2016, Prado e Castro et al. 2016, Weissmann et al. 2017, Rego et al. 2022).

### Step description

In the laboratory, the specimens were sorted and those classified as pollinators were later identified to the lowest taxonomic category. In a first step, pollinators were identified to family-level and then, using a reference collection and taxonomic literature, the specimens were assigned to species and deposited at the Dalberto Teixeira Pombo Insect Collection (DTP), University of the Azores.

## Geographic coverage

### Description

The study was carried out in Terceira Island (Azores Archipelago, Portugal) and covered three different habitat-types of this island (intensive pastures, semi-natural pastures and naturalised vegetation) (Fig. 2). The different habitat-types correspond to a gradient of human disturbance from intensive pastures (most disturbed) to naturalised vegetation (less disturbed). Thirty sampling sites (10 per habitat-type) were selected throughout the island to sample the pollinators (Table 1).

### Coordinates

 and 38.640 to 38.791 N latitude Latitude; and longitude -27.046 to -27.352 W Longitude.

## Taxonomic coverage

### Description

The study targeted the local flower visitors, specifically the insect groups often considered to be the most important pollinators, like bees, bumblebees, ants and wasps (Hymenoptera), butterflies and moths (Lepidoptera), beetles (Coleoptera) and larger-size flies (Diptera).

### Taxa included

**Table:** 

Rank	Scientific Name	Common Name
order	Coleoptera	Beetles
order	Diptera	Hoverflies, blowflies and other flies
order	Hymenoptera	Bees, bumblebees, wasps and ants
order	Lepidoptera	Butterflies and moths

## Temporal coverage

**Data range:** 2023-7-26 – 2023-9-07.

## Collection data

### Collection name

Entomoteca Dalberto Teixeira Pombo (DTP); University of the Azores.

### Collection identifier

DTP

### Specimen preservation method

All specimens were preserved in ethanol (96%).

## Usage licence

### Usage licence

Creative Commons Public Domain Waiver (CC-Zero)

## Data resources

### Data package title

Unveiling Azorean Pollinators: A Critical Step for Biodiversity and Conservation

### Resource link

https://doi.org/10.15468/bxh354

### Alternative identifiers

https://www.gbif.org/dataset/db765f95-20f4-49ef-8fe4-b57228200a2e

### Number of data sets

2

### Data set 1.

#### Data set name

Event table

#### Data format

Darwin Core Archive

#### Character set

UTF-8

#### Download URL

http://ipt.gbif.pt/ipt/resource?r=pollinators_terceira

#### Data format version

1.2

#### Description

The dataset was published in the Global Biodiversity Information Facility platform, GBIF (Boieiro et al. 2024a). The following data table includes all the records for which a taxonomic identification of the species was possible. The dataset submitted to GBIF is structured as a sample event dataset that has been published as a Darwin Core Archive (DwCA), which is a standardised format for sharing biodiversity data as a set of one or more data tables. The core data file contains 137 records (eventID). This GBIF IPT (Integrated Publishing Toolkit, Version 2.5.6) archives the data and, thus, serves as the data repository. The data and resource metadata are available for download in the Portuguese GBIF Portal IPT (Boieiro et al. 2024a).

**Data set 1.** 

Column label	Column description
id	Unique identification code for sampling event data.
type	The nature or genre of the resource, as defined by the Dublin Core standard. In our case "PhysicalObject".
datasetName	"Terceira Pollinator Inventory".
eventID	Identifier of the events, unique for the dataset.
samplingProtocol	The sampling protocol used to capture the species.
sampleSizeValue	The numeric amount of time spent in each sampling.
sampleSizeUnit	The unit of the sample size value.
eventDate	Range during which the record was collected.
year	The four-digit year in which the dwc:Event occurred, according to the Common Era Calendar.
month	The integer month in which the dwc:Event occurred.
day	The integer day of the month on which the dwc:Event occurred.
verbatimEventDate	The verbatim original representation of the date and time information for a dwc:Event. In this case, the season.
habitat	The habitat from which the sample was obtained.
locationID	Identifier of the location.
islandGroup	Name of archipelago, always Azores in the dataset.
island	Name of the island.
country	Country of the sampling site, always Portugal in the dataset.
countryCode	ISO code of the country of the sampling site, always PT in the dataset.
stateProvince	Name of the region of the sampling site, always Azores in the dataset.
municipality	Municipality of the sampling site.
locality	Name of the locality.
minimumElevationInMeters	The lower limit of the range of elevation (altitude, above sea level), in metres.
decimalLatitude	Approximate decimal latitude.
decimalLongitude	Approximate decimal longitude.
geodeticDatum	The ellipsoid, geodetic datum or spatial reference system (SRS), upon which the geographic coordinates given in decimalLatitude and decimalLongitude are based, always WGS84 in the dataset.
coordinateUncertaintyInMeters	Uncertainty of the coordinates of the centre of the sampling plot.
coordinatePrecision	Precision of the coordinates.
georeferenceSources	A list (concatenated and separated) of maps, gazetteers or other resources used to georeference the Location, described specifically enough to allow anyone in the future to use the same resources.

### Data set 2.

#### Data set name

Occurrence Table

#### Data format

Darwin Core Archive

#### Character set

UTF-8

#### Download URL

http://ipt.gbif.pt/ipt/resource?r=pollinators_terceira

#### Data format version

1.2

#### Description

The dataset was published in the Global Biodiversity Information Facility platform, GBIF (Boieiro et al. 2024a). The following data table includes all the records for which a taxonomic identification of the species was possible. The dataset submitted to GBIF is structured as an occurrence table that has been published as a Darwin Core Archive (DwCA), which is a standardised format for sharing biodiversity data as a set of one or more data tables. The core data file contains 1139 records (occurrenceID). This GBIF IPT (Integrated Publishing Toolkit, Version 2.5.6) archives the data and, thus, serves as the data repository. The data and resource metadata are available for download in the Portuguese GBIF Portal IPT (Boieiro et al. 2024a).

**Data set 2.** 

Column label	Column description
id	Unique identification code for sampling event data.
type	The nature or genre of the resource, as defined by the Dublin Core standard. In our case "PhysicalObject".
licence	Reference to the licence under which the record is published.
institutionID	The identity of the institution publishing the data.
collectionID	The identity of the collection publishing the data.
institutionCode	The code of the institution publishing the data.
collectionCode	The code of the collection where the specimens are conserved.
basisOfRecord	The nature of the data record.
dynamicProperties	Additional information about the process of the establishment of the species.
occurrenceID	Identifier of the record, coded as a global unique identifier.
recordedBy	A list (concatenated and separated) of names of people, groups or organisations who performed the sampling in the field.
organismQuantity	A number or enumeration value for the quantity of organisms.
organismQuantityType	The type of quantification system used for the quantity of organisms.
sex	The sex and quantity of the individuals captured.
lifeStage	The life stage of the organisms captured.
establishmentMeans	The process of establishment of the species in the location, using a controlled vocabulary: 'native', 'introduced', 'endemic', 'indeterminate'.
associatedTaxa	A list (concatenated and separated) of identifiers or names of dwc:Taxon records and the associations of this dwc:Occurrence to each of them. In this case, the plant host of the pollinator species.
eventID	Identifier of the events, unique for the dataset.
identifiedBy	A list (concatenated and separated) of names of people, groups or organisations who assigned the taxon to the subject.
dateIdentified	The date on which the subject was determined as representing the taxon.
scientificName	Complete scientific name including author and year.
kingdom	Kingdom name.
phylum	Phylum name.
class	Class name.
order	Order name.
family	Family name.
genus	Genus name.
specificEpithet	Specific epithet.
infraspecificEpithet	Infraspecific epithet.
taxonRank	Lowest taxonomic rank of the record.
scientificNameAuthorship	Name of the author of the lowest taxon rank included in the record.

## Additional information

### Results

In this study, we recorded 2547 individuals from 40 pollinator species in the three study habitat-types, using three complementary sampling techniques. Overall, we collected more than 6000 terrestrial arthropods, but many were by-catches resulting from unspecific sampling techniques (i.e. pan trapping and vegetation sweeping) (Boieiro et al. 2024a). The pollinator species recorded include coleopterans, dipterans, hymenopterans, lepidopterans and the introduced Madeiran wall lizard (*Teiradugesii*) (Table 2). Most species are native to the Azores, including three endemic taxa - the hoverflies *Sphaerophorianigra* and *Xanthandrusazorensis* and the butterfly *Pierisbrassicaeazorensis* - while 14 species are known to have been introduced in the Archipelago. Overall, the three habitat-types showed similar species richness values (ranging from 30 to 36) and most species (27 out 40) were found in all of them, irrespective of their distribution status (Table 2).

Despite the overall similarity in total species richness values between the study habitat-types, the findings obtained from the different sampling techniques were not consistent. Higher overall species richness values were found in the intensive pasture when using pan traps, while the findings from sweeping and transect observations showed higher species richness in naturalised vegetation (Fig. 3a). Larger differences in overall species richness between habitat-types were recorded when sampling by vegetation sweeping. Similar findings were found for overall pollinator abundance since the results obtained from the different sampling techniques were also variable. Higher overall pollinator abundance values were obtained in the intensive pasture when using pan traps, in the naturalised vegetation when using transect observations and in the semi-natural pastures following vegetation sweeping (Fig. 3b). Interestingly, marked differences in pollinator abundance between habitat-types were found when using pan traps.

**Figure 4.**
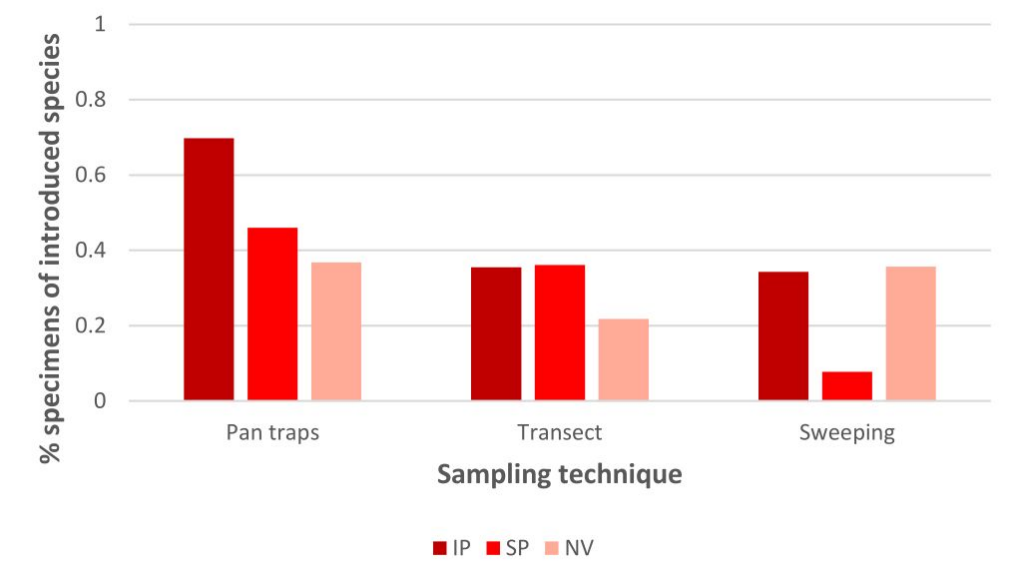
Percentage of specimens of introduced species in the three study habitat-types using different sampling techniques, namely pan trapping, observation transects and vegetation sweeping. The study habitat-types were Intensive Pasture (IP), Semi-natural Pasture (SP) and Naturalised Vegetation (NV).

The percentage of specimens of introduced species also changed between habitat-types. In general, this percentage increased along the disturbance gradient, with lowest values in the naturalised vegetation and highest in the intensive pasture (Fig. 4). However, a high percentage of specimens of introduced species was found in the samples of vegetation sweeping from the naturalised vegetation. This high value results from the high abundance of calliphorids and *Lasioglossum* bees, the latter being recently considered as probably introduced in the Azores (Weissmann et al. 2017, Borges et al. 2022a). Calliphorids and halictid bees were the most frequent and abundant specimens in pan traps and vegetation sweeping samples, particularly *Lasioglossum* spp., *Calliphora* spp. and *Stomorhinalunata*. Transect observations recorded a higher diversity of pollinator groups and species with several being frequent and/or abundant, like the butterflies *Coliascroceus* and *Pierisbrassicaeazorensis*, the hoverflies *Episyrphusbalteatus* and *Sphaerophoriascripta* and the apid bees *Apismellifera* and *Bombusterrestris* (Fig. 5).

### Discussion

Pollinators are essential for maintaining the biodiversity and stability of ecosystems and their regular monitoring is key to predict the effects of environmental changes on island communities and to timely assess changes in the pollination services that support food security. Our study advances understanding of Azorean pollinator communities by providing baseline data on species abundance, distribution and ecological associations. This information can help to guide landscape management and conservation efforts on Terceira Island.

In general, overall pollinator species richness showed minimal variation across the disturbance gradient, which ranged from naturalised vegetation to intensive pastures. This is most probably due to a combination of factors including the legacy of severe human disturbance of Azorean lowlands since human colonisation with the extirpation of native forests (and most probably the extinction of native specialist species) and their substitution by neobiota (Gaspar et al. 2008, Rego et al. 2015), the homogenisation of pollinator communities following the introduction, establishment and spread of many species from different taxonomic groups (Weissmann et al. 2017, Borges et al. 2022a) and the biophysical characteristics of the study habitats that, jointly with landscape structure, facilitate the dispersal of pollinators between habitat-types (Lazaro et al. 2016, Picanço et al. 2017b). However, overall pollinator species richness varied across habitat-types according to the sampling technique used since each of them introduces biases by favouring certain pollinator species/groups or their behaviour. Thus, the use of complementary sampling techniques is fundamental to provide a better understanding of pollinator communities since no single technique is able to capture the wide taxonomic, ecological and behavioural variety of this charismatic and diverse animal group (Roulston et al. 2007, Popic et al. 2013, O'Connor et al. 2019). Pan traps, observation transects and net sweeping are three common sampling techniques applied alone or in combination to sample pollinator communities. Pan traps are often considered effective for sampling some pollinators groups, particularly bees and their results are independent of observer expertise, but several authors have stressed their poor performance in sampling other pollinator groups (Westphal et al. 2008, O'Connor et al. 2019 and references therein). Observation transects are easy to implement and generate valuable ecological data, but their results may be influenced by observer expertise and biased towards more conspicuous species. Other sampling techniques, including net sweeping, are usually used to sample specific microhabitats and record cryptic pollinator species.

The overall percentage of specimens of alien species was higher in pastures than in the naturalised vegetation as recorded by pan traps and transect observations (Fig. 4). Various studies have reported that agricultural intensification and landscape simplification can lead to changes in the composition and structure of pollinator communities, generally benefitting introduced and more generalist species, alongside declines in the abundance, or even loss, of specialist and rare species (Weiner et al. 2014, Stein et al. 2018, Maurer et al. 2024). Our findings also match this pattern, but the differences are not so marked since the naturalised vegetation areas are also subjected to human disturbance (Picanço et al. 2017b). For example, introduced calliphorids (*Calliphora* spp., *Luciliasericata* and *Polleniarudis*) and bees (*Lasioglossum* spp.) are abundant and widespread in Azorean lowlands and seem to benefit from the simplified vegetation structure and moderate disturbance levels found in intensive pastures. The two Azorean endemic hoverflies - *Sphaerophorianigra* and *Xanthandrusazorensis* - are relatively common in the remnants of native forest located at higher altitudes, but were seldom observed in the more disturbed, lowland study areas. Nonetheless, most individuals of *X.azorensis* were found in areas with naturalised vegetation, where food resource availability is higher and the more complex vegetation structure offers protection from predators.

The study and conservation of island pollinators should be considered a priority due to their essential role in terrestrial ecosystems, as they enable the reproduction of numerous plants (including key crops), thereby supporting food security, biodiversity and ecosystem stability. It is, therefore, crucial to enhance our understanding on their abundance, distribution and ecology through standardised monitoring programmes, assess their extinction risks and develop conservation programmes to stop their decline and broadly share information on island pollinators to decision-makers, the research community and the public.

## Figures and Tables

**Figure 1a.**
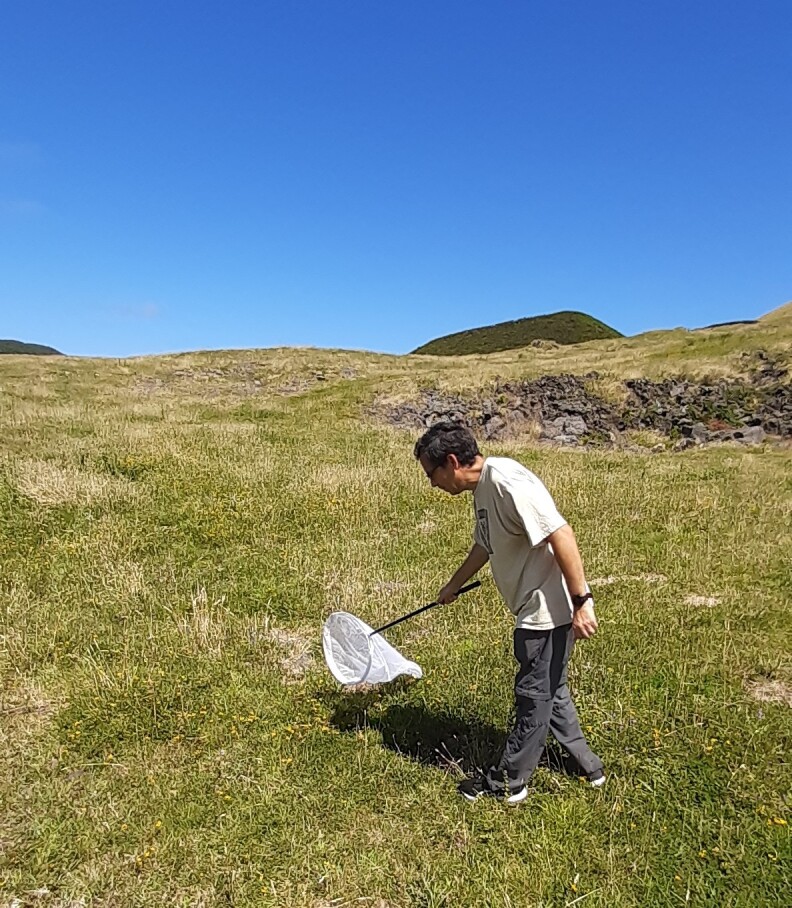
Vegetation sweeping along a transect (Credit: Raúl Oliveira);

**Figure 1b.**
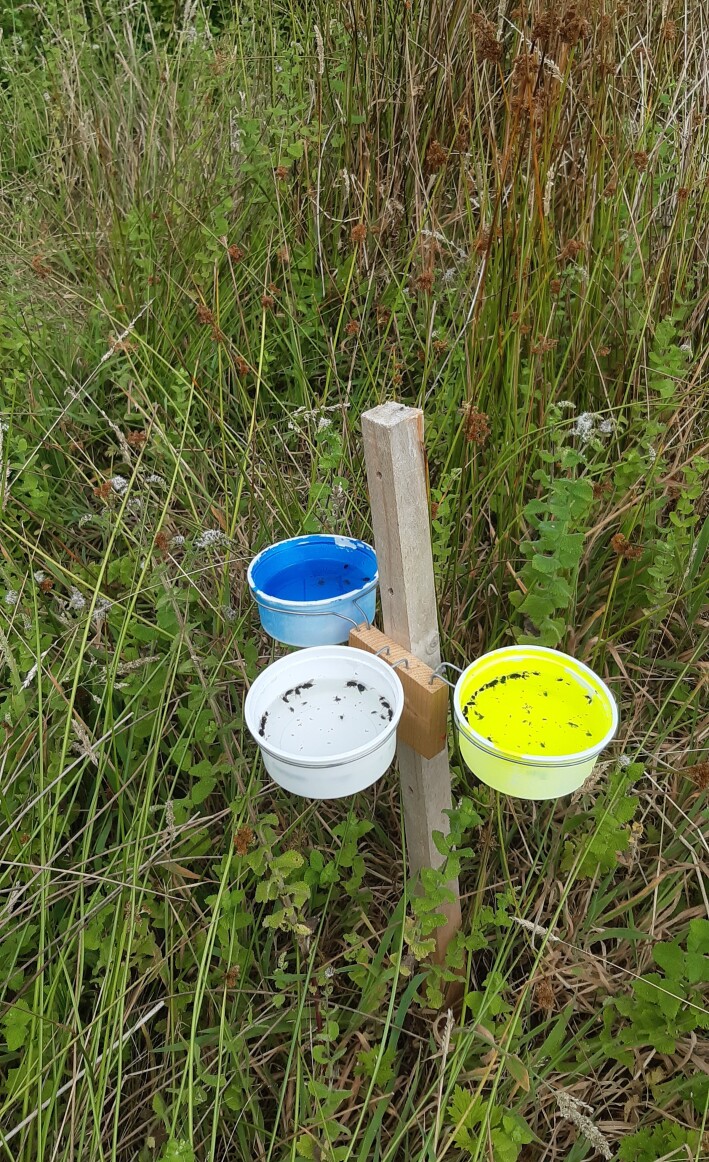
Pan trapping using a combination of three colour traps (Credit: Mário Boieiro).

**Figure 2a.**
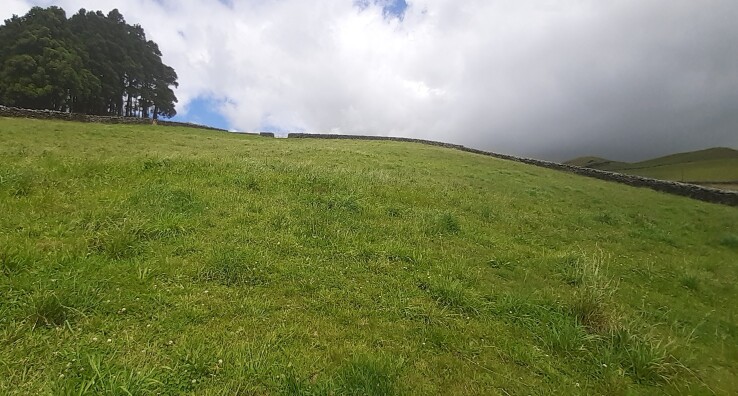
Intensive pastures (Credit: Mário Boieiro);

**Figure 2b.**
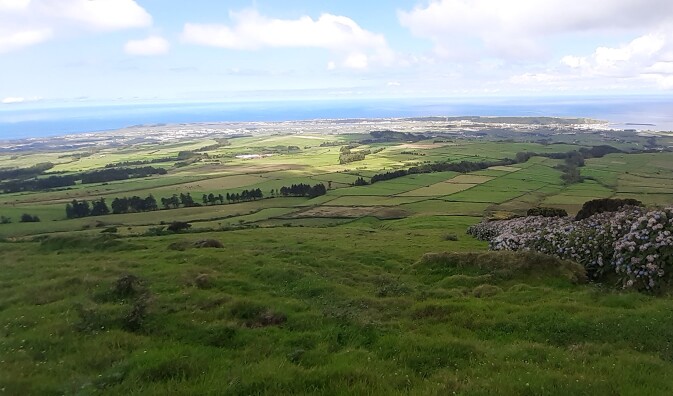
Semi-natural pastures (Credit: Mário Boieiro);

**Figure 2c.**
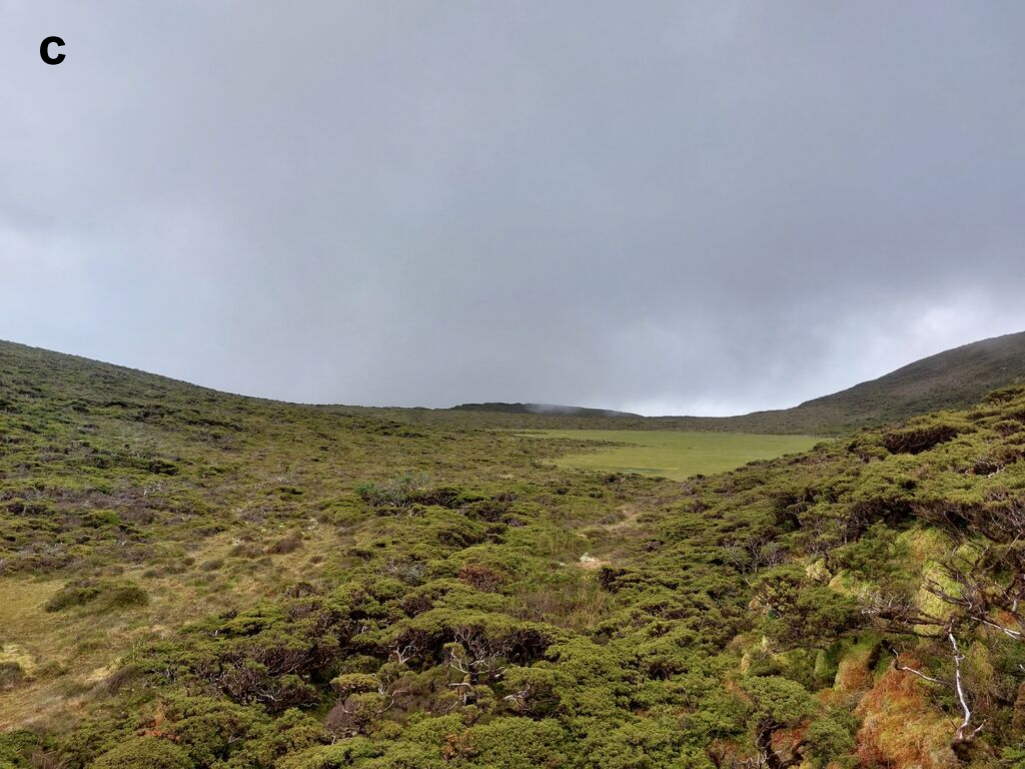
Areas of naturalised vegetation (Credit: Raúl Oliveira).

**Figure 3a.**
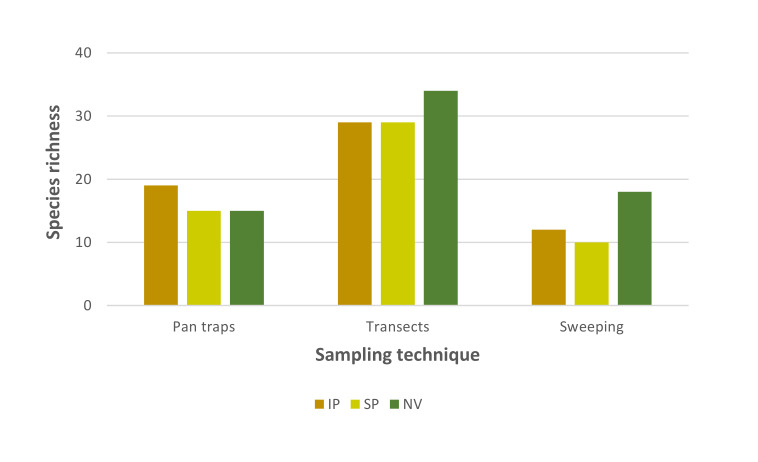
Overall pollinator species richness in the study habitat-types;

**Figure 3b.**
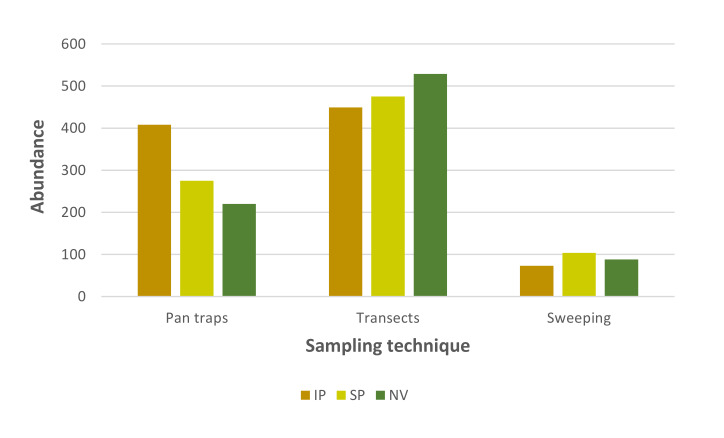
Overall pollinator abundance in the study habitat-types.

**Figure 5a.**
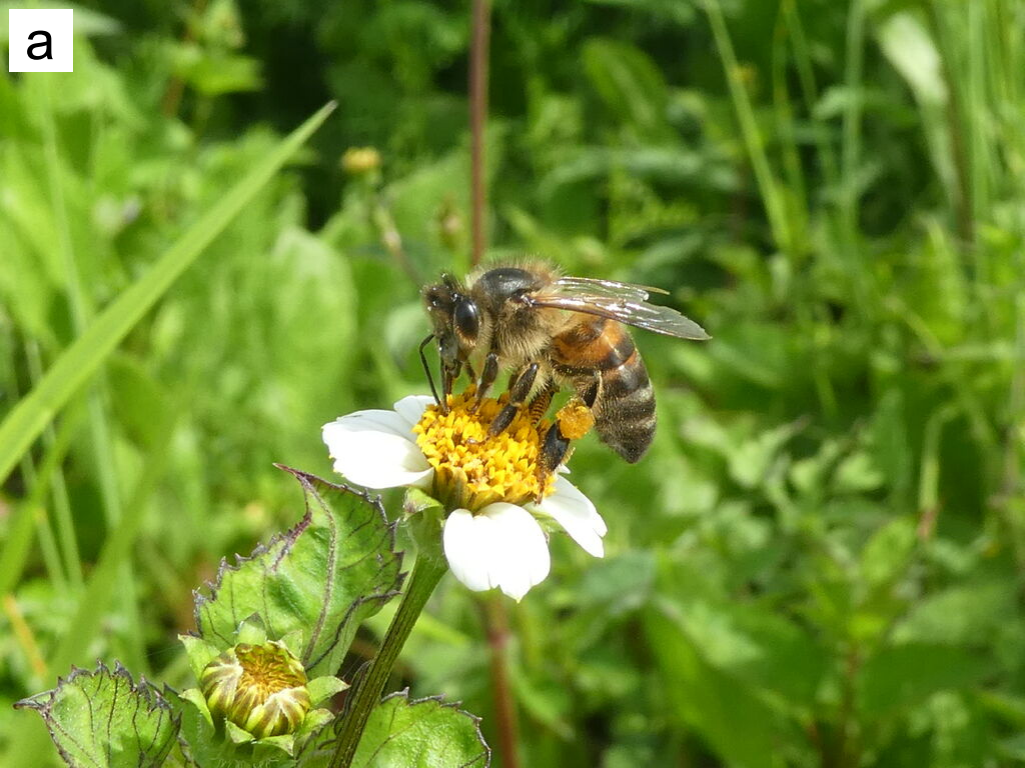
The introduced honeybee *Apismellifera* (Credit: Mário Boieiro);

**Figure 5b.**
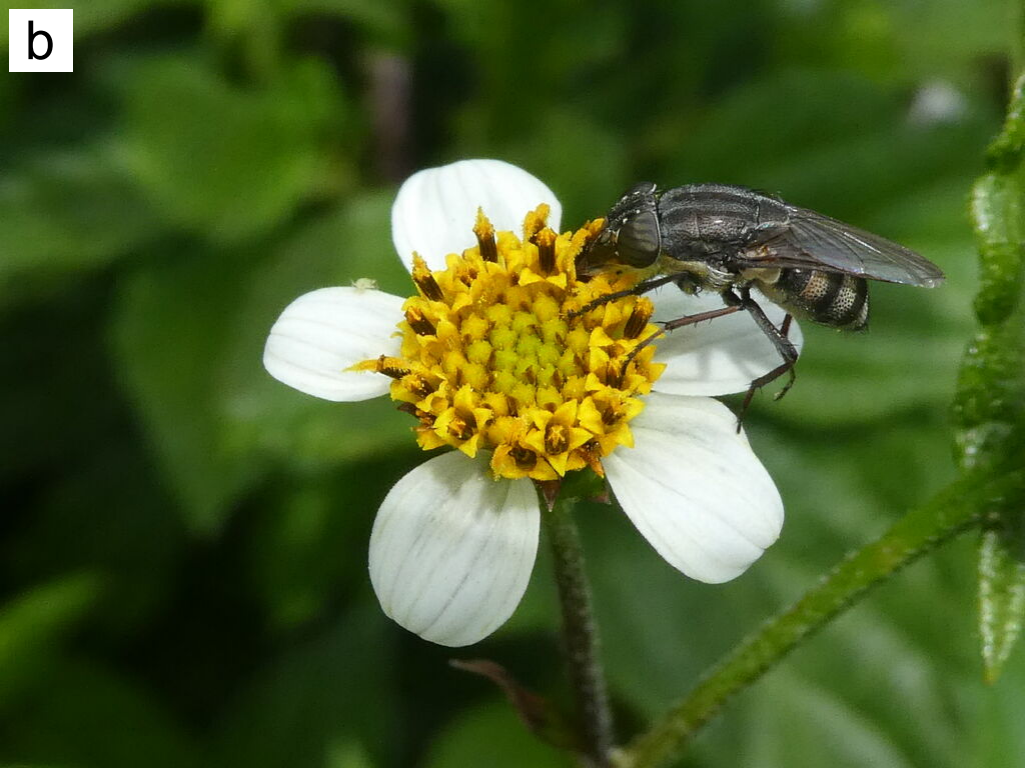
The native blowfly *Stomorrhinalunata* (Credit: Mário Boieiro);

**Figure 5c.**
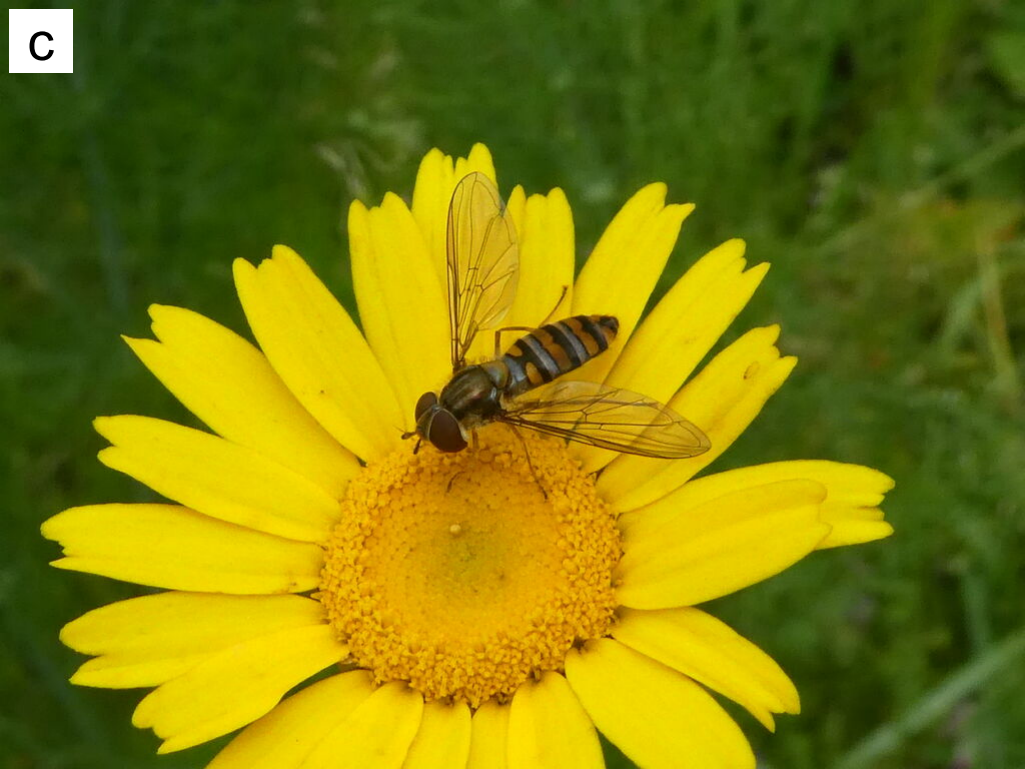
The native hoverfly *Episyrphusbalteatus* (Credit: Mário Boieiro);

**Figure 5d.**
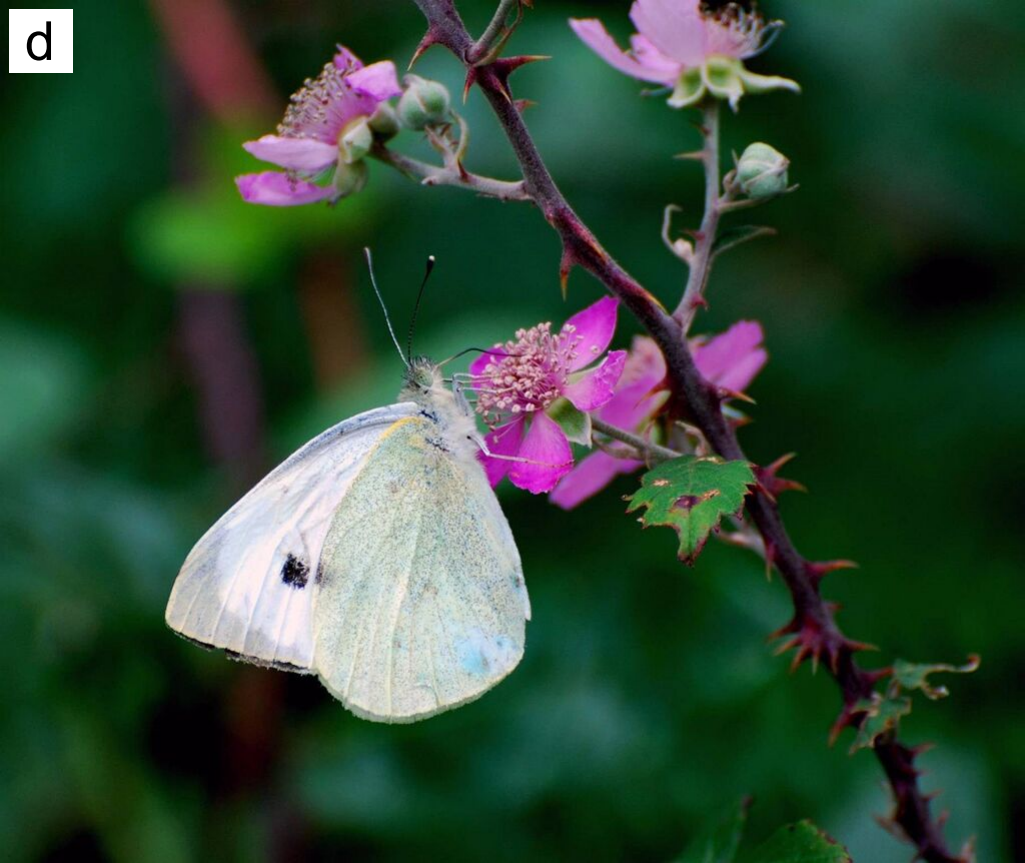
The endemic butterfly *Pierisbrassicaeazorensis* (Credit: Paulo A.V. Borges).

**Table 1.** List of the study sites with an indication of their location (in decimal degrees WGS84) and habitat-type.

**Site**	**Habitat-type**	**Latitude**	**Longitude**
São Bartolomeu	Intensive pasture	38.6798854	-27.2826606
Geodésico da Achada	Intensive pasture	38.7333167	-27.1367529
Vila Nova	Intensive pasture	38.7756620	-27.1448923
Vila de São Sebastião	Intensive pasture	38.6711610	-27.1124942
Altares	Intensive pasture	38.7735812	-27.2933906
Casa da Ribeira	Intensive pasture	38.7306579	-27.0907281
Cinco Ribeiras	Intensive pasture	38.6987552	-27.3045845
Altares	Intensive pasture	38.7871241	-27.2830835
Ribeira da Agualva	Intensive pasture	38.7540160	-27.1586589
Zona industrial	Intensive pasture	38.6927298	-27.1631230
Mata da Serreta	Naturalised vegetation	38.7653190	-27.3486071
Posto Santo	Naturalised vegetation	38.7223333	-27.2414148
Passagem das Bestas	Naturalised vegetation	38.7215142	-27.1976988
Agualva	Naturalised vegetation	38.7242645	-27.1677962
Juncal	Naturalised vegetation	38.7441911	-27.0677309
Alagoa	Naturalised vegetation	38.7900145	-27.1909792
Mata da Serreta - Lagoinha	Naturalised vegetation	38.7554467	-27.3347484
Algar do Carvão	Naturalised vegetation	38.7340980	-27.2161423
Monte Brasil	Naturalised vegetation	38.6439630	-27.2250641
Paúl da Pedreira	Naturalised vegetation	38.6485960	-27.1446360
Lagoa do Cerro	Semi-natural pasture	38.7501653	-27.2875407
Pico do Funil	Semi-natural pasture	38.7223953	-27.2092049
Serra do Cume	Semi-natural pasture	38.6927937	-27.1079285
Serra do Cume	Semi-natural pasture	38.7171179	-27.1160170
Serreta	Semi-natural pasture	38.7464084	-27.3439121
Cinco Ribeiras	Semi-natural pasture	38.7068506	-27.3103693
Doze Ribeiras	Semi-natural pasture	38.7220838	-27.3414948
Posto Santo	Semi-natural pasture	38.7178298	-27.2402493
Biscoitos	Semi-natural pasture	38.7538085	-27.2714432
Ganadaria Rego Botelho	Semi-natural pasture	38.7007910	-27.1953417

**Table 2.** List of pollinator species found in the study habitat-types (X - present, 0 - absent) and their distribution status in the Azores (following Borges et al. (2022a)). The study habitat-types were Intensive Pasture (IP), Semi-natural Pasture (SP) and Naturalised Vegetation (NV).

**Order**	**Family**	**Scientific name**	**Distribution status**	**Occurrence in habitat-types**		
				**IP**	**SP**	**NV**
Coleoptera	Nitidulidae	*Brassicogethesaeneus* (Fabricius, 1775)	Introduced	X	X	X
Coleoptera	Rutelidae	*Popilliajaponica* Newman, 1838	Introduced	X	X	X
Coleoptera	Scraptiidae	*Anaspisproteus* Wollaston, 1854	Native	X	0	X
Diptera	Calliphoridae	*Calliphoravicina* Robineau-Desvoidy, 1830	Introduced	X	X	X
Diptera	Calliphoridae	*Calliphoravomitoria* (Linnaeus, 1758)	Introduced	X	X	X
Diptera	Calliphoridae	*Luciliasericata* (Meigen, 1826)	Introduced	X	X	X
Diptera	Calliphoridae	*Polleniarudis* (Fabricius, 1794)	Introduced	X	X	X
Diptera	Calliphoridae	*Stomorhinalunata* (Fabricius, 1805)	Native	X	X	X
Diptera	Scathophagidae	*Scathophagastercoraria* (Linnaeus, 1758)	Native	X	X	X
Diptera	Syrphidae	*Episyrphusbalteatus* (De Geer, 1776)	Native	X	X	X
Diptera	Syrphidae	*Eristalisarbustorum* (Linnaeus, 1758)	Native	X	X	X
Diptera	Syrphidae	*Eristalistenax* (Linnaeus, 1758)	Native	X	X	X
Diptera	Syrphidae	*Eupeodescorollae* (Fabricius, 1794)	Native	X	0	X
Diptera	Syrphidae	*Melanostomamellinum* (Linnaeus, 1758)	Native	0	0	X
Diptera	Syrphidae	*Myathropaflorea* (Linnaeus, 1758)	Native	X	X	X
Diptera	Syrphidae	*Sphaerophorianigra* Frey, 1945	Endemic	X	X	X
Diptera	Syrphidae	*Sphaerophoriascripta* (Linnaeus, 1758)	Native	X	X	X
Diptera	Syrphidae	*Syrittapipiens* (Linnaeus, 1758)	Native	X	X	X
Diptera	Syrphidae	*Xanthandrusazorensis* Frey, 1945	Endemic	0	X	X
Diptera	Syrphidae	*Xylotasegnis* (Linnaeus, 1758)	Native	0	0	X
Diptera	Tachinidae	*Tachinafera* (Linnaeus, 1761)	Native	X	X	X
Hymenoptera	Apidae	*Apismellifera* Linnaeus, 1758	Introduced	X	X	X
Hymenoptera	Apidae	*Bombusruderatus* (Fabricius, 1775)	Introduced?	X	0	X
Hymenoptera	Apidae	*Bombusterrestris* (Linnaeus, 1758)	Introduced?	X	X	X
Hymenoptera	Crabronidae	*Pemphredon* sp.	Native	X	0	0
Hymenoptera	Formicidae	*Lasiusgrandis* Forel 1909	Native	X	X	X
Hymenoptera	Formicidae	*Monomoriumcarbonarium* (Smith, 1858)	Native	0	X	0
Hymenoptera	Halictidae	*Lasioglossumlativentre* (Schenk, 1853)	Introduced?	X	X	X
Hymenoptera	Halictidae	*Lasioglossummalachurum* (Kirby, 1802)	Introduced?	X	X	X
Hymenoptera	Halictidae	*Lasioglossummorio* (Fabricius, 1793)	Introduced?	X	X	X
Hymenoptera	Halictidae	*Lasioglossumvillosulum* (Kirby, 1802)	Native?	X	X	X
Hymenoptera	Megachilidae	*Megachilecentuncularis* (Linnaeus, 1758)	Introduced?	X	X	X
Hymenoptera	Vespidae	*Ancistrocerusgazella* (Panzer, 1798)	Native	X	X	X
Hymenoptera	Vespidae	*Ancistrocerusparietum* (Linnaeus, 1758)	Native	0	0	X
Lepidoptera	Lycaenidae	*Lampidesboeticus* (Linnaeus, 1767)	Native	0	X	X
Lepidoptera	Nymphalidae	*Vanessaatalanta* (Linnaeus, 1758)	Native	X	0	X
Lepidoptera	Pieridae	*Coliascroceus* (Fourcroy, 1785)	Native	X	X	X
Lepidoptera	Pieridae	*Pierisbrassicaeazorensis* Rebel, 1917	Endemic	X	X	X
Lepidoptera	Sphingidae	*Macroglossumstellatarum* (Linnaeus, 1758)	Native	X	0	0
Squamata	Lacertidae	*Teiradugesii* (Milne-Edwards, 1829)	Introduced	X	0	0
